# Supplementary material for: Effects of Ti Target Purity and Microstructure on Deposition Rate, Microstructure and Properties of Ti Films
Source: Materials (Basel). 2022 Apr 5;15(7):2661. doi: 10.3390/ma15072661 (PMC9000231; doi:10.3390/ma15072661)
Supplement: Supplementary file 1 [file materials-15-02661-s001.zip › materials-1640137-supplementary.pdf]

materials

Supplementary Information

Effects of Ti Target Purity and Microstructure on Deposition Rate, Microstructure and Properties of Ti Films

Elemental Analysis of Ti Targets of Different Purity are as follows.

Table S1. Elemental composition of high purity Ti target.

Ti target (99.999%) Elements Analysis (ppm)								
Li	Be	B	Na	Mg	Al	Si	P	S
< 0.01	< 0.005	< 0.01	< 0.01	0.06	0.11	0.24	< 0.01	0.02
K	Ca	Sc	V	Cr	Mn	Fe	Co	Ni
< 0.01	< 0.2	< 0.05	0.02	0.06	0.06	3.2	< 0.01	0.24
Cu	Zn	Ga	Ge	As	Se	Zr	Nb	Mo
0.28	< 0.05	< 0.05	< 0.05	0.03	< 0.05	< 0.05	< 0.2	< 0.5
Ru	Rh	Pd	Ag	Cd	In	Sn	Sb	Te
< 0.01	< 0.05	< 0.01	< 0.05	< 0.05	< 0.05	< 0.05	< 0.05	< 0.05
Ba	Ce	Hf	Ta	F	Cl	Br	I	Cs
< 0.005	< 0.005	< 0.01	< 5	< 0.05	0.23	< 0.05	< 0.01	< 0.01
La	Nd	Sm	Dy	Er	W	Re	Os	Ir
< 0.005	< 0.005	< 0.005	< 0.005	< 0.005	< 0.01	< 0.01	< 0.01	< 0.01
Pt	Au	Hg	Pb	Bi	Th			
< 0.05	< 0.05	< 0.1	< 0.01	< 0.01	< 0.0001			

Table S2. Elemental composition of industrial Ti target.

Ti target (99.99%) Elements Analysis (wt %)							
Mg	Al	Ca	Si	Cr	Mn	Fe	Co
< 0.0001	< 0.0002	< 0.0001	< 0.0005	< 0.0001	< 0.0005	< 0.0002	< 0.0001
Cu	Zn	As	Se	Mo	Pb	Cd	In
0.0002	< 0.0001	< 0.0001	< 0.0003	< 0.0001	< 0.0005	< 0.0001	< 0.0002
Sb	Te	Ba	Pr	Ir	Au	Pb	Bi
< 0.0005	< 0.0005	< 0.0001	< 0.0005	< 0.0002	< 0.0005	< 0.0001	< 0.0005
Ni	Sn						
< 0.0002	< 0.0005						

Table S3. Elemental composition of low purity Ti target.

Ti target (99.7%) Elements Analysis (wt %)									
C	N	H	O	Fe	Cu	Cr	Ag	Mg	Mn
0.015	0.01	0.01	0.01	0.05	0.002	0.005	0.002	0.001	0.001
